# *Saxifraga damingshanensis* (*S.* sect. *Irregulares*, Saxifragaceae), a new species from Guangxi, China

**DOI:** 10.3897/phytokeys.133.36704

**Published:** 2019-10-16

**Authors:** Wan-Yi Zhao, Kai-Kai Meng, Qiang Fan, Jian-Hua Jin, Wen-Bo Liao

**Affiliations:** 1 State Key Laboratory of Biocontrol and Guangdong Provincial Key Laboratory of Plant Resources, School of Life Sciences, Sun Yat-sen University, Guangzhou 510275, China Sun Yat-sen University Guangzhou China

**Keywords:** China, chloroplast gene, phylogeny

## Abstract

*Saxifraga damingshanensis* (Saxifragaceae), a new species from Damingshan Nature Reserve in Guangxi Province, is described and illustrated. A morphological comparison between the new species and its putative relatives, *S. mengtzeana* and *S. luoxiaoensis*, is presented. The new species is morphologically similar to *S. mengtzeana*, but it can be easily distinguished by its non-peltate leaf, both surfaces of mature leaf blade covered with white glandular trichome, petals 3-veined and margin entire. Phylogenetic analysis, based on two chloroplast DNA regions (*matK* and *psbA-trnH*), confirmed that the new species belongs to S. sect. Irregulares. The new species is currently only known from Damingshan, Guangxi and we assign it an IUCN Red List preliminary status as Data Deficient.

## Introduction

*Saxifraga* L. (Saxifragaceae) is widely distributed throughout the Northern Hemisphere and comprises ca. 440–450 species (Pan et al. 2001; Tkach et al. 2015a, b). Numerous previous molecular phylogenetic studies suggested that *Saxifraga* is monophyletic, providing that S. sect. Micranthes (Haw.) D.Don is excluded and the genus *Micranthes* Haw. recognised. (Soltis et al. 1996; Prieto et al. 2013; Deng et al. 2015; Tkach et al. 2015a, b). Saxifraga sect. Irregulares Haw., characterised by long-petiolate leaves, leafless flowering stems with small bracts, stamens with club-shaped filaments and pollen grains with numerous microchannels in the tectum, is the earliest-diverging named clade in *Saxifraga* (Soltis et al. 2001; Zhang et al. 2015; Tkach et al. 2015b).

In China, *Saxifraga* comprises 216 species, of which seven species belong to S. sect. Irregulares, according to the "Flora of China" (Pan et al. 2001). Recently, some new species of S. sect. Irregulares were discovered in China, including *S. daqiaoensis* F.W.Wang & F.W.Xing (Wang et al. 2008), *S. kegangii* D.G.Zhang, Y.Meng & M.H.Zhang (Zhang et al. 2017), *S. luoxiaoensis* W.B.Liao, L.Wang & X.J.Zhang (Zhang et al. 2018) and *S. shennongii* L.Wang, W.B.Liao & J.J.Zhang (Zhang et al. 2019).

During a botanical expedition to Damingshan National Nature Reserve, Wuming district, central Guangxi Province in September 2018, we discovered an unknown species of *Saxifraga* in Longtou Peak. Its mature leaves are densely covered with white trichomes and the abaxial surface is densely purple-spotted. After carefully checking specimens and literature, as well as morphological and molecular studies, we confirm that it is a new species of *Saxifraga* and it is described below.

## Materials and methods

We collected more than 20 living individuals of the presumed new species for comparisons and taxonomical treatment. Specimens of Saxifraga sect. Irregulares, available at herbaria (PE, IBSC, SYS and IBK) and digital photos of all herbarium specimens of *S. luoxiaoensis*, *S. mengtzeana* Engl. & Irmsch., preserved in the Chinese Virtual Herbarium (http://www.cvh.org.cn/), have been checked. Five main characters (leaf shape, leaf margin, spots on the abaxial surface of leaf, petal shape and trichomes on plants) of these three species were compared both in the wild and in the herbarium.

To determine the systematic position of *Saxifraga damingshanensis*, we further sampled five individuals of the presumed new species for a phylogenetic study. The geographic sampling information of these individuals was recorded by a Garmin GPS unit (GPSMAP 62sc, Taiwan) and the voucher specimens were deposited at Sun Yat-sen University Herbarium (**SYS**) (Table 1). The final molecular dataset comprises 19 accessions representing eight species of S. sect. Irregulares, of which five accessions were newly generated and 14 accessions were downloaded from GenBank (Table 1).

**Table 1.** Voucher information and GenBank accession numbers for sequence data of *Saxifraga damingshanensis* phylogenetic analysis used in this study.

Species	Voucher	*matK*	*psbA-trnH2*
***Saxifraga damingshanensis***	**W. Y. Zhao 1208; Damingshan, Guangxi**	**MK976729**	**MK976724**
	**W. Y. Zhao 1209; Damingshan, Guangxi**	**MK976730**	**MK976725**
	**W. Y. Zhao 1210; Damingshan, Guangxi**	**MK976731**	**MK976726**
	**W. Y. Zhao 1211; Damingshan, Guangxi**	**MK976732**	**MK976727**
	**W. Y. Zhao 1212; Damingshan, Guangxi**	**MK976733**	**MK976728**
*Saxifraga mengtzeana*	FHZ-1608; Yuanbao Mountion, Guangxi	MK092518	–
*Saxifraga rufescens*	YLDP197D; Yulong Mountion, Yunnan	MH116857	MH117313
*Saxifraga stolonifera*	LXP-13- 24775(1); Yanling country, Hunan	MK092557	MK092599
	LXP-13- 24775(2); Yanling country, Hunan	MK092558	MK092600
	LXP-13- 24775(3); Yanling country, Hunan	MK092551	MK092593
	LXP-13- 24775(4); Yanling country, Hunan	MK092552	MK092594
*Saxifraga epiphylla*	Q. Fan 15680(1); Qingchengshan, Sichuan	MK092519	–
	Q. Fan 15680(2); Qingchengshan, Sichuan	MK092520	–
*Saxifraga daqiaoensis*	RY-2017-031(1); Daqiao Town, Guangdong	MK092533	MK092575
	RY-2017-031(2); Daqiao Town, Guangdong	MK092534	MK092576
*Saxifraga luoxiaoensis*	LXP-13-24717(1); Nanfengmian, Jiangxi	MK092539	MK092581
	LXP-13-24717(2); Nanfengmian, Jiangxi	MK092540	MK092582
*Saxifraga shennongii*	LXP-13-24778(1); Yanling country, Hunan	MK092527	MK092569
	LXP-13-24778(2); Yanling country, Hunan	MK092528	MK092570
	LXP-13-24769(1); Yanling country, Hunan	MK092521	MK092563
	LXP-13-24769(2); Yanling country, Hunan	MK092522	MK092564

*All vouchers are deposited in the Sun Yat-sen University Herbarium (SYS); “/” represents missing data.

The total DNA was extracted with the modified CTAB method (Doyle and Doyle 1987). The *psbA-trnH2* and *matK* intergenic regions were amplified using previously reported primers (Tate and Simpson 2003; Zhang et al. 2019). PCR amplifications were performed following Chen et al. (2016). Sequences were aligned with MEGA version 6.0 and subsequently manually adjusted (Tamura et al. 2013). Phylogenetic reconstructions were carried out with Maximum Likelihood (ML) and Bayesian Inference (BI) analyses. ML was run by IQ-Tree 1.6.10 with 20,000 ultrafast bootstraps and SH-like approximate likelihood ratio test (aLRT) of 10,000 replicates (Nguyen et al. 2015). BI was executed in MrBayes version 3.2 (Ronquist et al. 2012), with four chains for at least 20,000,000 generations to make the average standard deviation of split frequencies (ASDFs) < 0.01, sampling every 1000 generations with the first 25% sampled trees discarded as burn-in. The 50% majority-rule consensus trees were finally generated. For both ML and BI analyses, F81+F+I was detected as the best-fitting nucleotide substitution model on the basis of Bayesian Information Criterion (BIC) detected by ModelFinder (Kalyaanamoorthy et al. 2017).

## Results

### Morphological comparison

In morphology, the putative new species is closely related to *Saxifraga mengtzeana* and their morphology comparisons are presented in Table 2. These two species share such features as having stolons absent, inflorescences and pedicels covered with glandular hairs, white flowers without pink markings and base of three short petals with yellow plot. However, the new species differs from *S. mengtzeana* by having leaf base cordate to deep cordate (vs. usually peltate), leaves papery or nearly leathery (vs. leathery), adaxial surface of the mature leaf with glandular trichome (*vs.* nearly glabrous) and longest petal 3-veined, margin entire (vs. 8-veined, margin sparsely denticulate). Moreover, *S. damingshanensis* flowers from August to October, while *S. mengtzeana* flowers from March to August.

**Table 2.** Morphological comparisons amongst *Saxifraga damingshanensis*, *S. luoxiaoensis* and *S. mengtzeana*.

Characters	*Saxifraga damingshanensis*	*S. luoxiaoensis*	*S. mengtzeana*
Leaf shape	rounded or ovate, never peltate	reniform, never peltate	± peltate, ovate
Leaf texture	papery or leathery	papery	leathery
Leaf base	cordate to deep cordate	cordate	± cordate
Leaf margin	5–10-lobed, lobes entire, sparsely glandular hairy	margin 7–9-lobed, usually double serrate	crenate, inconspicuous glandular hairy
Abaxial surface of leaf blade	with glandular trichome and purple spots	glabrous with red or brown spotted	sparsely hispid and brown spotted
Second longest petal	lanceolate oblong, 13–17 × 2 mm, 3-veined	lanceolate oblong, ca. 8–20 mm × 1.3–3 mm, 3−5-veined	narrowly ovate, ca. 9 × 2.2 mm, 3-veined
First longest Petal	lanceolate, 1.8–2.2 cm × 1.5–2.5 mm, 3-veined, margin entire	linear lanceolate, 16–25 mm × 1.3 − 3 mm, 3–5-veined, margin entire	sublanceolate, 19 × 3.4 mm, 8-veined, margin sparsely denticulate
Stamens	3.5–4.5 mm long	4.3–5.6 mm long	ca. 6 mm long
Flowering period	August to October	April to June	May to August

### Phylogenetic placement of *Saxifraga damingshanensis* within S. sect. Irregulares

**Figure 1.**
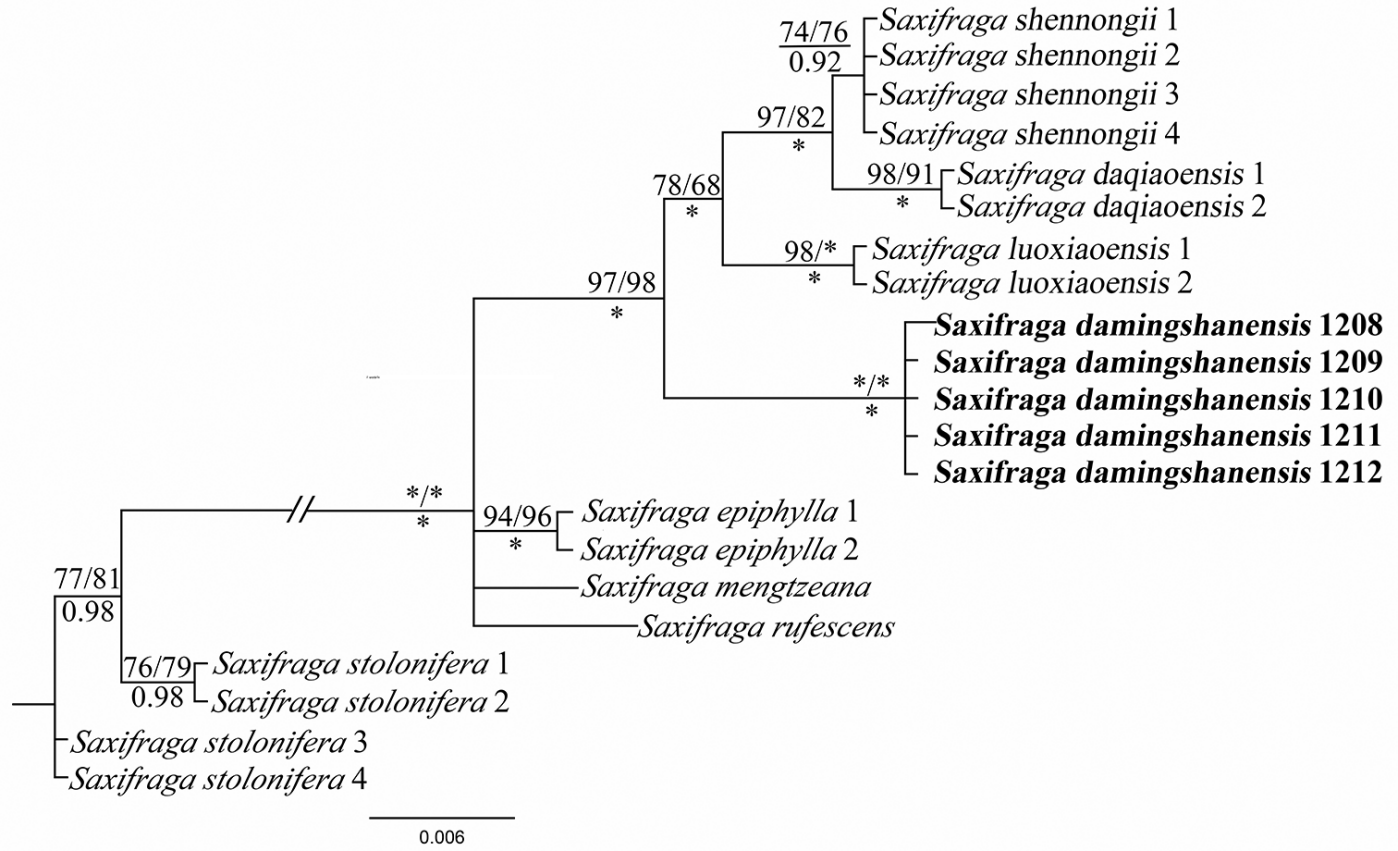
Bayesian consensus tree of *Saxifraga damingshanensis* and related species derived from two chloroplast regions. Numbers above branches are the value of SH-like approximate likelihood ratio test (aLRT) and bootstrap value of the Maximum Likelihood (LP); numbers below branches indicate Bayesian posterior probability (PP). Asterisks denoted (*) the values of 100 or 1.00 for LP/PP. The new species is shown in bold.

The concatenated sequences of *matK* (740 bp) and *psbA-trnH2* (297 bp) are 1037 bp in length and 81 parsimony-informative sites were detected. Our results showed that S. ser. Rufescentes J.T.Pan is monophyletic (SH-aLRT: 100; LP: 100; PP: 1.00, Fig. 1) which is coincident with the previous study (Zhang et al. 2018). The putative new species, *S. damingshanensis*, was nested into S. ser. Rufescentes J.T.Pan and was strongly supported as sister to a clade consisting of *S. luoxiaoensis*, *S. daqiaoensis* and *S. shennongii* (SH-aLRT: 97; LP: 98; PP: 1.0).

## Discussion

Based on its basal leaves with long petiolate, flower zygomorphic and stamens with club-shaped filaments, the new species could be placed within S. sect. Irregulares. Our phylogeny also supports the inclusion of *Saxifraga damingshanensis* within S. sect. Irregulares (Fig. 1). All examined individuals of *S. damingshanensis* clustered into a single lineage, thus corroborating the evidence for the new species status, based on morphology.

Our study also recovered a sister relationship of the new species with a clade comprising *Saxifraga luoxiaoensis*, *S. daqiaoensis* and *S. shennongii*. The close relationship amongst these species was also supported by their similar morphological characteristics. All four species have white glandular trichomes on leaf and inflorescence and white and entire petals. However, *S. damingshanensis* differs from the latter three species by having mature leaf and petiole with glandular trichomes (vs. mature leaf sparsely hispid or glabrous) and the abaxial surface of the leaf blade with purple spots (vs. usually yellow-brown spots). Furthermore, their phenology and distribution are different. *Saxifraga damingshanensis* is flowering from August to October (vs. April to June) and endemic to Damingshan, Guangxi (*vs.* Guangdong, Jiangxi and Hunan).

### Taxonomic treatment

#### Saxifraga damingshanensis

Taxon classificationPlantaeSaxifragalesSaxifragaceae

W.B.Liao, W.Y.Zhao & J.H.Jin sp. nov.

738678EE-48E0-5C44-BE10-583F1AF5B3AB

urn:lsid:ipni.org:names:77202383-1

##### Type.

**Figure 2.**
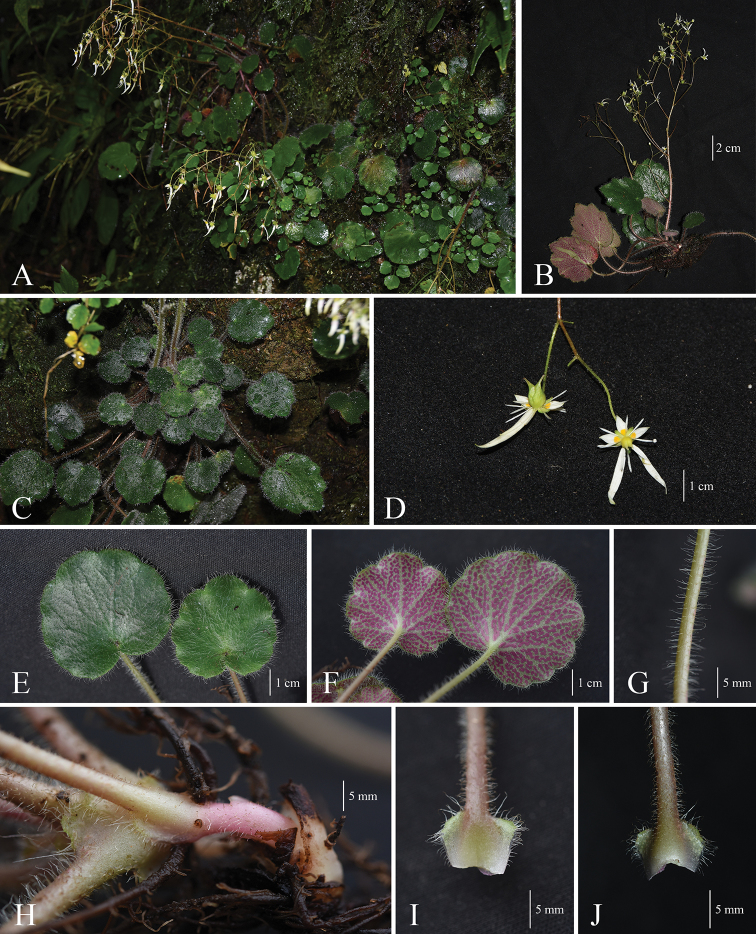
*Saxifraga damingshanensis***A** Habit **B** whole plant **C** basal leaves rosette with long petiole, plant cover white trichomes **D** flower and fruit, pedicel slender with short trichomes, filaments clavate **E** adaxial leaf surface dark green, sparsely glandular piliferous **F** abaxial leaf surface grey, sparsely glandular piliferous and purple spotted **G** petiole with glandular piliferous **H** rhizomes cover sparsely glandular piliferous, petiole base sheathed **I** adaxial surface of sheath, glabrous, margin with glandular piliferous **J** abaxial surface of sheath, upper with sparse glandular piliferous.

China. Guangxi: Nanning city, Damingshan Nature Reserve, Longtou Peak, 23°22'58.48"N, 108°30'21.56"E, 1542 m alt., 19 September 2018, *W.Y.Zhao 1208* (Holotype SYS!; Isotypes SYS!, IBSC!). (Fig. 2)

##### Diagnosis.

*Saxifraga damingshanensis* is morphologically most similar to *S. mengtzeana*, but differs by its leaf blade with glandular trichome and purple spots abaxially, short stamens and petal entire.

##### Description.

Perennial herbs, 15–30 cm tall. Stolons absent. Rhizomes rather short (ca. 0.3–0.7 cm), sparsely glandular trichomes. Basal leaves forming a rosette, petiole 5–15 cm long, fleshy and translucent, sparsely short glandular trichomes (ca. 2 mm); petiole base sheathed, margin with sparsely glandular trichomes; leaf blade rounded or ovate, base cordate to deep cordate, papery or leathery, 2.0–5.7 × 2.5–5.5 cm, apex obtuse, margin inconspicuous 7–15-lobed with sparsely glandular trichomes, lobes entire, adaxially dark green, densely covered glandular trichomes (2.5–4 mm), abaxially grey, sparsely covered with glandular trichomes (1.5–2.5 mm), densely covered with purple spots; palmate veins 7–11, both surfaces inconspicuous. Cauline leaves 1–2, triangular-lanceolate, 5.0–6.0 × 1.5–2.0 mm, margin with sparsely glandular trichomes. Inflorescence paniculate, 15–30 cm long, 10–35-flowered; branches 4–5 cm, sparsely short glandular trichomes (0.5–1.0 mm), 2–6(-8)-flowered; pedicels slender, 1.5–2.5 cm long, sparsely short glandular trichomes (ca. 0.5 mm); bracts linear, 1.5–2.5 × 0.5–0.8 mm, margin with short glandular trichomes. Flowers zygomorphic; sepals 5, spreading, narrowly ovate, 2.0–2.5 × 1.0 mm, apex obtuse, base connate, adaxially glabrous, abaxial surface and margin with sparsely short glandular trichome, becoming denser proximally, 3 veins inconspicuous. Petals 5, white; shortest 3 petals equal, ovate, base with yellow spots, 3–3.5 × 1.5 mm, apex acute, base rounded, triplinerved; the other two petals lanceolate, first longest petal lanceolate, 18–22 × 1.5–2.5 mm, apex acuminate, margin entire, glabrous, 3-veined; second longest petal narrowly ovate, 1.3–1.7 × 0.2 cm, apex acuminate, margin entire, glabrous, 3-veined. Stamens 10, filaments clavate, 3.5–4.5 mm long. Ovary ovoid, 1.5–2.5 mm long; disc obscure; carpels 2, proximally connate about 3/4; styles 2, divergent, 1.5–2 mm long. Capsule ovoid, 4–5 × 3–4 mm. Seeds elongate-ellipsoid, yellowish-brown or dark brown, the two ends slightly bent, ca. 0.6 mm, surface 3-ribbed.

##### Phenology.

Flowering from August to October, fruiting from September to November.

##### Etymology.

The species epithet is based on the mountain name, Damingshan and the Latin suffix, -*ensis*, of origin, where the new species was collected.

##### Distribution, ecology and conservation status.

Only three populations of *Saxifraga damingshanensis* were discovered from Damingshan National Nature Reserve, Guangxi Province. It was observed to grow on damp cliffs and rocks in broad-leaved forests at altitudes between 1300 and 1650 m. Its known localities are well protected and more field investigations are needed to determine its wild distribution. Therefore, we suggest listing the new species as Data Deficient (DD) based on the IUCN Red List Criteria (IUCN 2019).

##### Additional specimens examined (paratypes).

China. Guangxi: Nanning city, Damingshan Nature Reserve, Longtou Peak, Blackwood cliff, 23°22'58.48"N, 108°30'21.56"E, 1542 m alt., 19 September 2018, *W.Y.Zhao 1209* (SYS!); same locality, 1500 m alt., 19 September 2018, *W.Y.Zhao 1210* (SYS!); same locality, 1522 m alt., 19 September 2018, *W.Y.Zhao 1211* (SYS!); same locality,1480 m alt., 19 September 2018, *W.Y.Zhao 1212* (SYS!); Wuming county, Xiaolu village, Damingshan, 1420 m alt., 26 August 1958, *Y.C.Chen 325* (IBK18155!).

## Supplementary Material

XML Treatment for Saxifraga damingshanensis
